# Prognostic value of inflammatory markers for in-hospital mortality in intensive care patients with acute ischemic stroke: a retrospective observational study based on MIMIC-IV

**DOI:** 10.3389/fneur.2023.1174711

**Published:** 2023-06-08

**Authors:** Xuyang Hu, Jiaru Liang, Wenjian Hao, Jiaqi Zhou, Yuling Gao, Xiaoyang Gong, Yong Liu

**Affiliations:** ^1^Department of Rehabilitation Medicine, The First Affiliated Hospital of Dalian Medical University, Dalian, China; ^2^Institute (College) of Integrative Medicine, Dalian Medical University, Dalian, China

**Keywords:** acute ischemic stroke, in-hospital mortality, MIMIC-IV, SII, inflammatory marker, predictor

## Abstract

**Background:**

Acute ischemic stroke (AIS) is a primary cause of death and disability worldwide. Four markers that can be readily determined from peripheral blood, namely, the systemic immune-inflammation index (SII), neutrophil-to-lymphocyte ratio (NLR), platelet-to-lymphocyte ratio (PLR), and total bilirubin, were measured in this study. We examined the relationship between the SII and in-hospital mortality after AIS and evaluated which of the above four indicators was most accurate for predicting in-hospital mortality after AIS.

**Methods:**

We selected patients from the Medical Information Mart for Intensive Care-IV (MIMIC-IV) database who were aged >18 years and who were diagnosed with AIS on admission. We collected the patients’ baseline characteristics, including various clinical and laboratory data. To investigate the relationship between the SII and in-hospital mortality in patients with AIS, we employed the generalized additive model (GAM). Differences in in-hospital mortality between the groups were summarized by the Kaplan–Meier survival analysis and the log-rank test. The receiver operating characteristic (ROC) curve analysis was used to assess the accuracy of the four indicators (SII, NLR, PLR, and total bilirubin) for predicting in-hospital mortality in patients with AIS.

**Results:**

The study included 463 patients, and the in-hospital mortality rate was 12.31%. The GAM analysis showed a positive correlation between the SII and in-hospital mortality in patients with AIS, but the correlation was not linear. Unadjusted Cox regression identified a link between a high SII and an increased probability of in-hospital mortality. We also found that patients with an SII of >1,232 (Q2 group) had a considerably higher chance of in-hospital mortality than those with a low SII (Q1 group). The Kaplan–Meier analysis demonstrated that patients with an elevated SII had a significantly lower chance of surviving their hospital stay than those with a low SII. According to the results of the ROC curve analysis, the in-hospital mortality of patients with AIS predicted by the SII had an area under the ROC curve of 0.65, which revealed that the SII had a better discriminative ability than the NLR, PLR, and total bilirubin.

**Conclusion:**

The in-hospital mortality of patients with AIS and the SII were positively correlated, but not linearly. A high SII was associated with a worse prognosis in patients with AIS. The SII had a modest level of discrimination for forecasting in-hospital mortality. The SII was slightly better than the NLR and significantly better than the PLR and total bilirubin for predicting in-hospital mortality in patients with AIS.

## Introduction

Stroke, which is the third leading cause of disability and the second leading cause of mortality worldwide, is a common and damaging disease, and the majority of stroke cases are ischemic stroke due to arterial occlusive disease (1). According to data from China’s Hospital Quality Monitoring System, 2,466,785 patients with ischemic stroke were hospitalized in 2018, accounting for 81.9% of all stroke cases, placing a huge burden on society (2). Therefore, it is of great significance to identify convenient and efficient biomarkers to predict disease prognosis, which could reduce the adverse outcomes of patients with stroke.

The mechanisms of acute ischemic stroke (AIS) are complex and multifactorial. Scientific evidence links inflammation, which exacerbates brain damage, to the occurrence, progression, and outcome of AIS (3, 4). During the early stages of ischemic stroke, peripheral immune populations, including neutrophils, monocytes, T cells, and macrophages, infiltrate the brain parenchyma (5). Therefore, the assessment of inflammatory indicators is helpful to evaluate the prognosis of AIS. It is well known that AIS is closely associated with many inflammatory markers, including interleukin, high-sensitivity C-reactive protein, tumor necrosis factor, and homocysteine, amongst others (6, 7). In addition to the abovementioned indicators, several composite inflammatory markers have been used to predict the prognosis of patients with AIS. The neutrophil-to-lymphocyte ratio (NLR), which reflects the balance between circulating neutrophils and lymphocytes, is strongly associated with short-term functional outcomes in patients with AIS (8). Moreover, the platelet-to-lymphocyte ratio (PLR) is a strong predictor of AIS prognosis and could be used to assess platelet activation due to inflammation-coagulation interactions and other factors (9, 10). The systemic-immune inflammation index (SII) is also associated with poor outcomes in patients with AIS, reflecting thrombotic and immune dysregulation (11). Furthermore, once cerebral ischemia occurs, excessive oxidative stress ensues, resulting in structural and functional damage to the brain (12). As two early events of cerebral ischemic injury, inflammation and oxidative stress are closely related (13). Moreover, bilirubin is the most effective endogenous antioxidant and plays a neuroprotective role in stroke. Many studies have revealed a correlation between bilirubin and poor outcomes in patients with AIS, but there is still some controversy (12, 14–16).

The abovementioned four markers (SII, NLR, PLR, and bilirubin) can be readily determined from peripheral blood and are strongly associated with poor outcomes in patients with AIS. Therefore, this study sought to investigate the relationship between the SII and in-hospital mortality in intensive care patients with AIS and to examine which of these four inflammatory markers is most effective at predicting short-term mortality from AIS.

## Methods

### Medical information mart for intensive care-IV (MIMIC-IV) database

This retrospective and observational study was conducted based on primary data obtained from the comprehensive MIMIC-IV database. MIMIC-IV comprises numbers for each medical record relating to patients who were admitted to the intensive care unit (ICU) or emergency room at the Beth Israel Deaconess Medical Center between 2008 and 2019 (17). The first author (Xuyang Hu, certification ID: 51415516) was authorized to use the MIMIC-IV database after completing the National Institutes of Health’s online education program. The BIDMC Institutional Review Board assessed the gathering of patient data and the development of the research resource, authorized the data-sharing project, and waived the requirement for informed consent. To ensure patient privacy, all processes were completed in compliance with the applicable regulations.

### Patient selection

Using the International Classification of Diseases (ICD) codes ICD-9: 433, ICD-9: 434, ICD-9: 436, and ICD-10: I63, we selected 1,605 patients who were admitted to the ICU and who were diagnosed with AIS from the MIMIC-IV database. The inclusion criteria were as follows: (I) patients aged >18 years; (II) patients diagnosed with AIS; and (III) patients admitted to the ICU. The exclusion criteria were as follows: (I) patients with incomplete or difficult-to-find documentation or other important medical records; (II) patients with missing survival outcome data; and (III) patients with missing data on white blood cell count, neutrophil count, lymphocyte count, platelet count, or bilirubin concentration.

### Patients’ baseline characteristics

Patients’ baseline characteristics were collected, including general information, vital signs, comorbidity history, laboratory parameters, and scoring system results. We extracted the first record of various data for patients diagnosed with AIS on admission. The vital signs included heart rate, mean blood pressure, systolic blood pressure, diastolic blood pressure, respiratory rate, body temperature, and pulse oximetry-derived oxygen saturation (SpO_2_). The anion gap, blood urea nitrogen, bicarbonate, creatinine, chloride, glucose, hematocrit, hemoglobin, bilirubin, chloride, neutrophil count, serum sodium, lymphocyte count, serum potassium, prothrombin time, white blood cell count, and platelet count were among the laboratory parameters that were recorded.

Both the Sequential Organ Failure Assessment (SOFA) score and Simplified Acute Physiology Score (SAPS) II for each patient were also calculated. In-hospital mortality was the endpoint of the study, as assessed by in-hospital survival.

The formula used to determine the SII was SII = platelet × neutrophil count ÷ lymphocyte count. The neutrophil count divided by the lymphocyte count was used to determine the NLR, while the platelet count divided by the lymphocyte count was used to determine the PLR.

### Statistical analysis

We used the generalized additive model (GAM) to examine the association between the SII and in-hospital mortality in patients with AIS. According to the GAM analysis results, the patients were divided into two groups. Normally distributed continuous variables are presented as the mean ± standard deviation, whereas non-normally distributed continuous variables are presented as the median. Categorical variables are expressed as frequency and percentage. The groups were compared using the chi-square test, Kruskal–Wallis test, and one-way analysis of variance. Differences in in-hospital mortality between the groups were summarized using the Kaplan–Meier survival analysis and the log-rank test. Due to the possibility of confounding effects of variables based on laboratory tests and epidemiology, we utilized three quartile-based Cox proportional hazards regression models, the first of which was used as the reference model.

We adjusted the covariates of comorbidities and vital sign data, including age, sex, heart rate, mean blood pressure, SpO_2_, congestive heart failure, renal failure, and temperature, in Model I. Model II was mostly modified for laboratory data, including creatinine, anion gap, hemoglobin, prothrombin time, glucose, and chloride. Based on Model II, the variables were further modified for the severity of illness scoring (SOFA score, SAPS II). Through the receiver operating characteristic (ROC) curve analysis, the discriminative ability of the four inflammatory indicators (SII, NLR, PLR, and total bilirubin) for predicting in-hospital mortality in patients with AIS was determined using the area under the ROC curve (AUC). Discrimination was considered good if the AUC exceeded 0.7 and moderate if the AUC was between 0.65 and 0.70. Statistical information was displayed as hazard ratios (HRs) and 95% confidence intervals (CIs). Each statistical test was conducted using a two-tailed design. R version 4.2.2 was used for the statistical analysis, and a *p* value of ≤ 0.05 was considered statistically significant.

## Results

### Baseline characteristics

In total, 463 suitable patients with a mean age of 71.68 ± 16.29 years (221 men and 242 women) were included in the study. Supplementary Tables S1, S2 provide further details on the data extraction procedure and missing data. The study yielded an in-hospital mortality rate of 12.31%, with 57 patients dying during hospitalization. According to the findings of the GAM analysis of the SII and in-hospital mortality, the patients were divided equally into two groups. In Table 1, the baseline characteristics of the groups are broken down according to the SII. The values for temperature, anion gap, blood glucose, and SAPS II were higher among patients with a high SII.

**Table 1:** Patients’ characteristics.

Characteristic	Total (*n* = 463)	Q1 (*n* = 231)	Q2 (*n* = 232)	*p*-value
Age (years)	71.68 ± 16.29	71.58 ± 15.80	71.77 ± 16.81	0.901
Male	221 (47.73%)	117 (50.65%)	104 (44.83%)	0.246
SBP (mmHg)	134.63 ± 18.20	135.32 ± 18.43	133.97 ± 17.99	0.424
DBP (mmHg)	73.66 ± 12.22	73.68 ± 12.32	73.64 ± 12.14	0.972
MBP (mmHg)	90.53 ± 12.46	90.89 ± 12.42	90.17 ± 12.52	0.534
Heart rate (beats/min)	79.32 ± 14.98	78.46 ± 14.64	80.17 ± 15.30	0.218
Respiratory rate (breaths/min)	19.31 ± 3.12	19.37 ± 3.34	19.25 ± 2.89	0.671
Temperature (°C)	36.94 ± 0.34	36.90 ± 0.32	36.97 ± 0.35	**0.044**
SpO_2_ (%)	96.78 ± 1.91	96.79 ± 2.03	96.78 ± 1.79	0.935
**Comorbidities, *n* (%)**				
Diabetes mellitus	151 (32.61%)	80 (34.63%)	71 (30.60%)	0.389
Myocardial infarction	57 (12.31%)	32 (13.85%)	25 (10.78%)	0.386
Congestive heart failure	119 (25.70%)	52 (22.51%)	67 (28.88%)	0.144
Chronic pulmonary disease	60 (12.96%)	29 (12.55%)	31 (13.36%)	0.904
Dementia	40 (8.64%)	21 (9.09%)	19 (8.19%)	0.857
Renal disease	89 (19.22%)	51 (22.08%)	38 (16.38%)	0.150
Malignancy	36 (7.78%)	20 (8.66%)	16 (6.90%)	0.593
**Laboratory parameters**				
Anion gap (mEq/L)	14.92 ± 3.10	14.56 ± 3.18	15.27 ± 2.98	**0.014**
BUN (mg/dL)	21.05 ± 15.15	21.05 ± 13.75	21.05 ± 16.45	0.999
Bicarbonate (mmol/L)	22.86 ± 3.14	23.05 ± 3.17	22.68 ± 3.12	0.210
Creatinine (mg/dL)	1.19 ± 1.34	1.26 ± 1.47	1.12 ± 1.20	0.260
Chloride (mmol/L)	103.51 ± 4.53	103.94 ± 4.40	103.70 ± 4.62	**0.038**
Glucose (mg/dL)	131.88 ± 47.24	126.82 ± 44.59	136.92 ± 49.32	**0.021**
Hematocrit (%)	37.20 ± 5.80	37.40 ± 5.88	37.00 ± 5.73	0.466
Hemoglobin (g/dL)	12.12 ± 2.12	12.18 ± 2.13	12.06 ± 2.10	0.533
Total bilirubin (mg/dL)	1.21 (2.26)	1.50 (2.82)	0.91 (1.46)	**0.005**
Neutrophil count (10^9^/L)	7.66 ± 3.92	5.79 ± 2.56	9.52 ± 4.15	<0.001
Lymphocyte count (10^9^/L)	1.67 ± 2.34	2.21 ± 3.19	1.13 ± 0.53	<0.001
Platelet count (10^9^/L)	211.00 ± 83.00	196 ± 66.00	231.00 ± 90.00	<0.001
Potassium (mmol/L)	4.24 ± 0.53	4.24 ± 0.54	4.24 ± 0.53	0.979
PT (s)	13.34 ± 3.99	13.10 ± 3.42	13.57 ± 4.49	0.205
Sodium (mmol/L)	139.91 ± 3.88	140.20 ± 3.78	139.61 ± 3.97	0.101
WBC count (10^9^/L)	10.28 ± 4.53	9.45 ± 5.00	10.36 ± 4.02	0.702
SOFA score	3.54 ± 0.06	3.75 ± 3.03	3.39 ± 2.53	0.165
SAPS II	32.64 ± 11.89	31.67 ± 11.21	33.61 ± 12.48	0.078
NLR	5.08 ± 6.75	3.62 ± 3.50	10.26 ± 7.54	<0.001
SII	1084.24 ± 1628.66	628.67 ± 259.37	2369.75 ± 1928.50	<0.001
ICU LOS (days)	4.55 ± 5.00	4.66 ± 4.86	4.45 ± 5.14	0.646
HOS LOS (days)	10.55 ± 11.61	10.18 ± 10.50	10.92 ± 12.64	0.494
In-hospital mortality	57 (12.31%)	20 (8.66%)	37 (15.95%)	0.025

SBP, systolic blood pressure; DBP, diastolic blood pressure; MBP, mean blood pressure; SpO_2_, pulse oximetry-derived oxygen saturation; BUN, blood urea nitrogen; PT, prothrombin time; WBC, white blood cell; SOFA, Sequential Organ Failure Assessment; SAPS II, Simplified Acute Physiology Score II; NLR, neutrophil-to-lymphocyte ratio; SII, systemic immune-inflammation index; ICU, intensive care unit; HOS, hospital; LOS, length of stay.

The data are presented as the mean ± standard deviation and median or *n* (%).

The bold values for temperature, anion gap, blood glucose, and SAPS II were higher among patients with a high SII.

### Relationship between the SII and in-hospital mortality in patients with AIS

**Figure 1:**
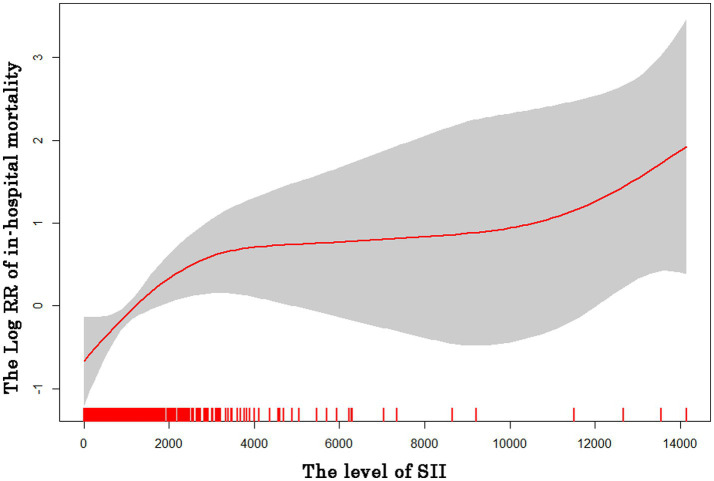
Cubic spline plot of relation of SII to risk of in-hospital patients’ mortality.

**Figure 2:**
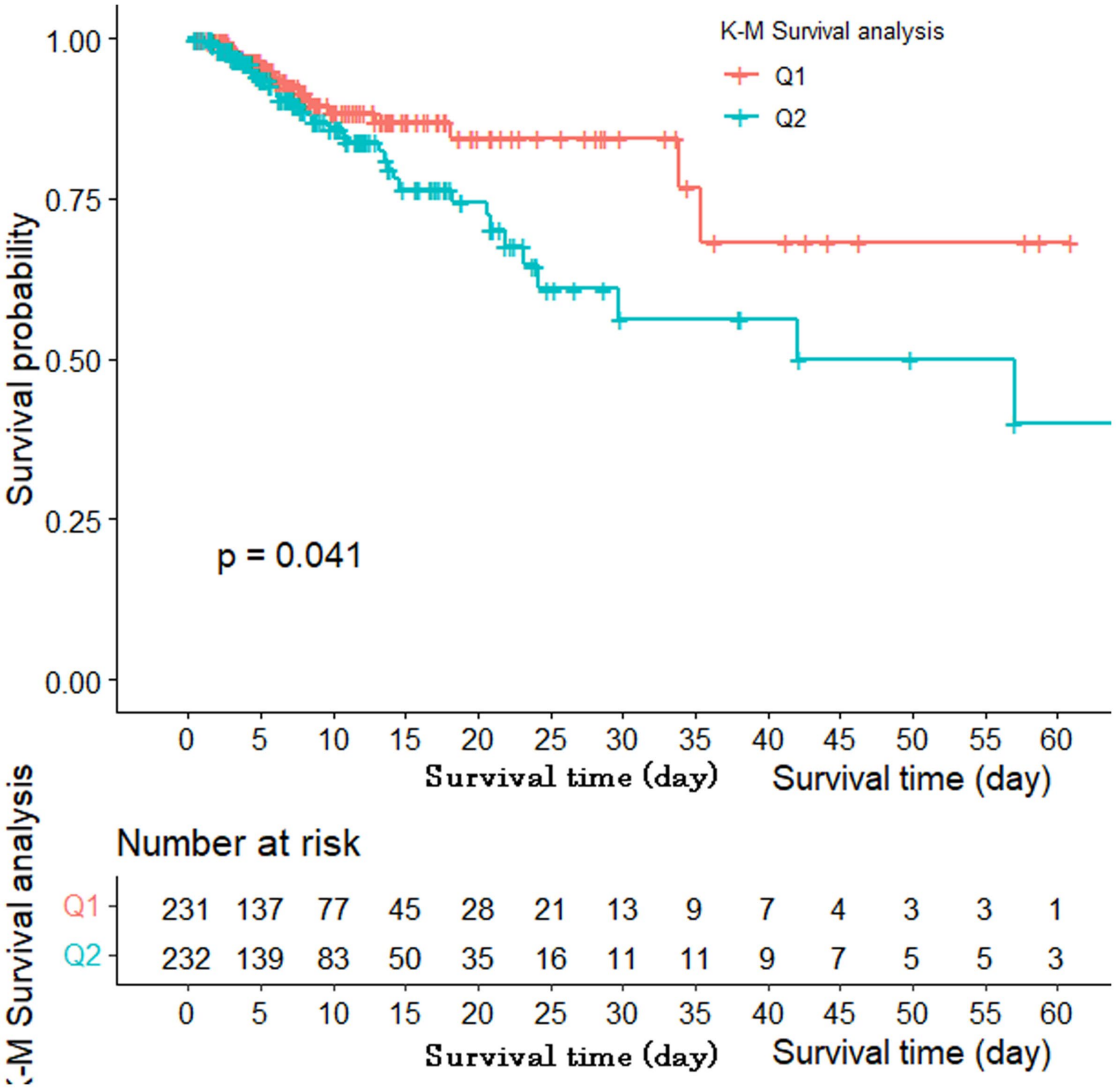
Kaplan–Meier survival curve of SII.

According to the results of the GAM analysis, the in-hospital mortality of patients with AIS was positively correlated with the SII, but not linearly (Figure 1). A high SII was associated with a higher risk of in-hospital mortality according to the unadjusted Cox regression analysis (HR 1.75, 95% CI 1.02–3.02, *p* = 0.044). We further explored the relationship between the SII and in-hospital mortality in patients with AIS using three Cox regression models to account for the influence of other confounding variables (Table 2). After adjusting for vital signs and comorbidities, a high SII was associated with increased in-hospital mortality in Model I (HR 1.97, 95% CI 1.13–3.44, *p* = 0.016). After adjusting for laboratory data on the basis of Model I, the HR of high SII was 1.96 in Model II (95% CI 1.11–3.48, *p* = 0.020). Based on Model II, the variables were further modified in Model III for the severity of illness scoring (SAPS II and SOFA score). The high SII group still had a considerably higher likelihood of in-hospital mortality (HR 2.06, 95% CI 1.15–3.72, *p* = 0.016). The Kaplan–Meier survival plot for patients with various SII values is shown in Figure 2. The results demonstrate that patients with an elevated SII had a significantly lower chance of surviving their hospital stay than those with a low SII (log-rank test: *p* = 0.041).

**Table 2:** The SII and in-hospital mortality of patients with AIS.

Variable	Unadjusted model		Model I		Model II		Model III	
SII	HR (95% CI)	*p*-value	HR (95% CI)	*p*-value	HR (95% CI)	*p*-value	HR (95% CI)	*p*-value
Q1	1.0 (ref)		1.0 (ref)		1.0 (ref)			
Q2	1.75 (1.02–3.02)	0.044	1.97 (1.13–3.44)	0.016	1.96 (1.11–3.48)	0.020	2.06 (1.15–3.72)	0.016

HR, hazard ratio; SII, systemic immune-inflammation index.

Model I was adjusted for age, sex, heart rate, mean blood pressure, SpO_2_, congestive heart failure, renal failure, and temperature.

Model II was adjusted for the variables adjusted in Model I, as well as creatinine, anion gap, hemoglobin, prothrombin time, glucose, and chloride.

Model III was adjusted for the variables adjusted in Model I and Model II, as well as the SAPS II and SOFA scores.

### The discriminative ability of the SII to predict in-hospital mortality in patients with AIS compared with the other indicators

**Figure 3:**
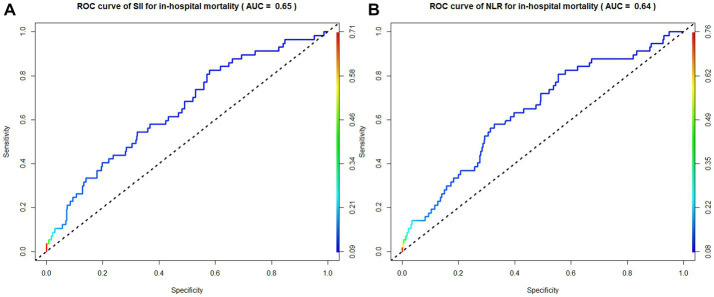
Receiver operating characteristic (ROC) curve of SII and NLR.

**Figure 4:**
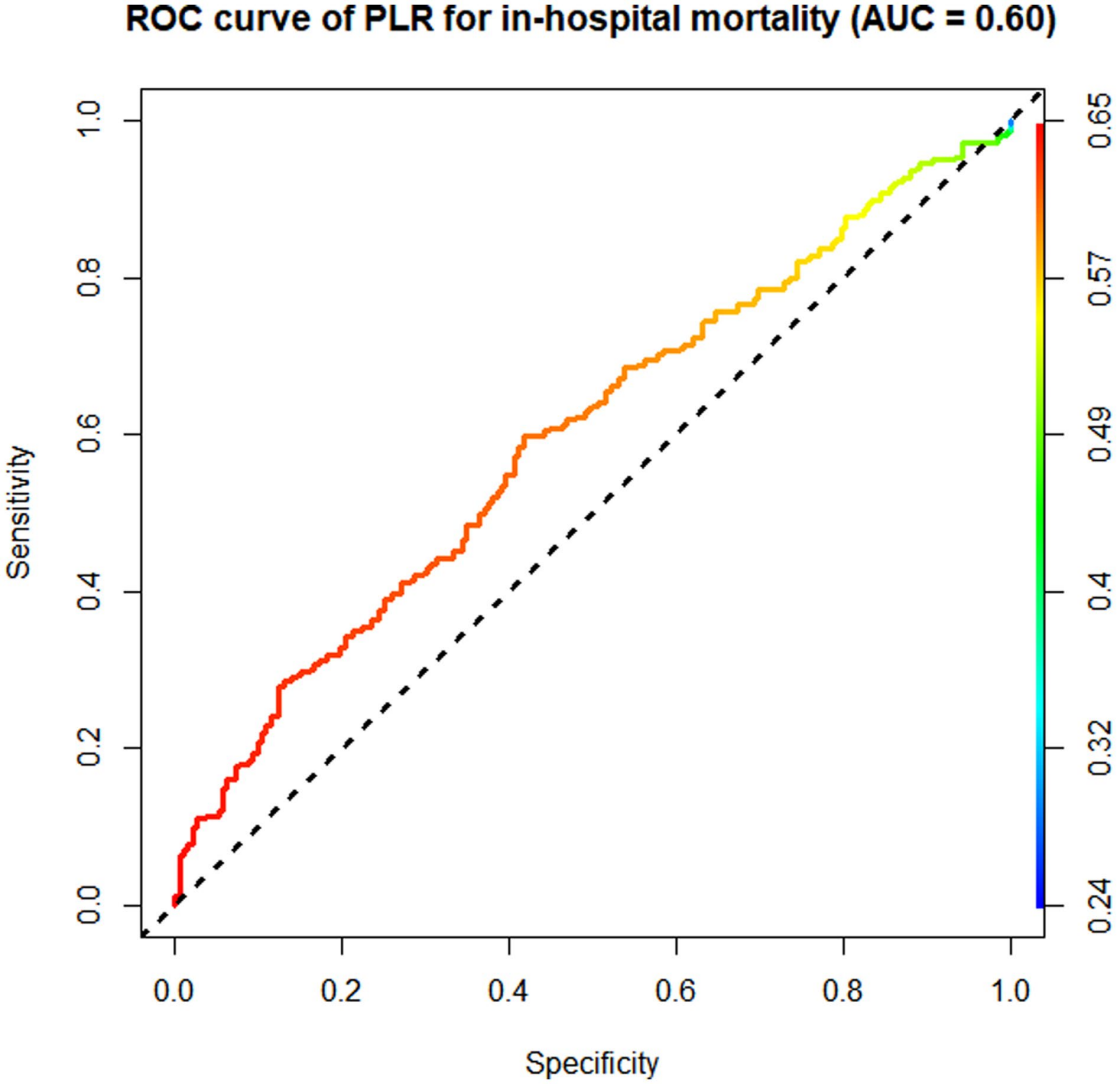
Receiver operating characteristic (ROC) curve of PLR.

**Figure 5:**
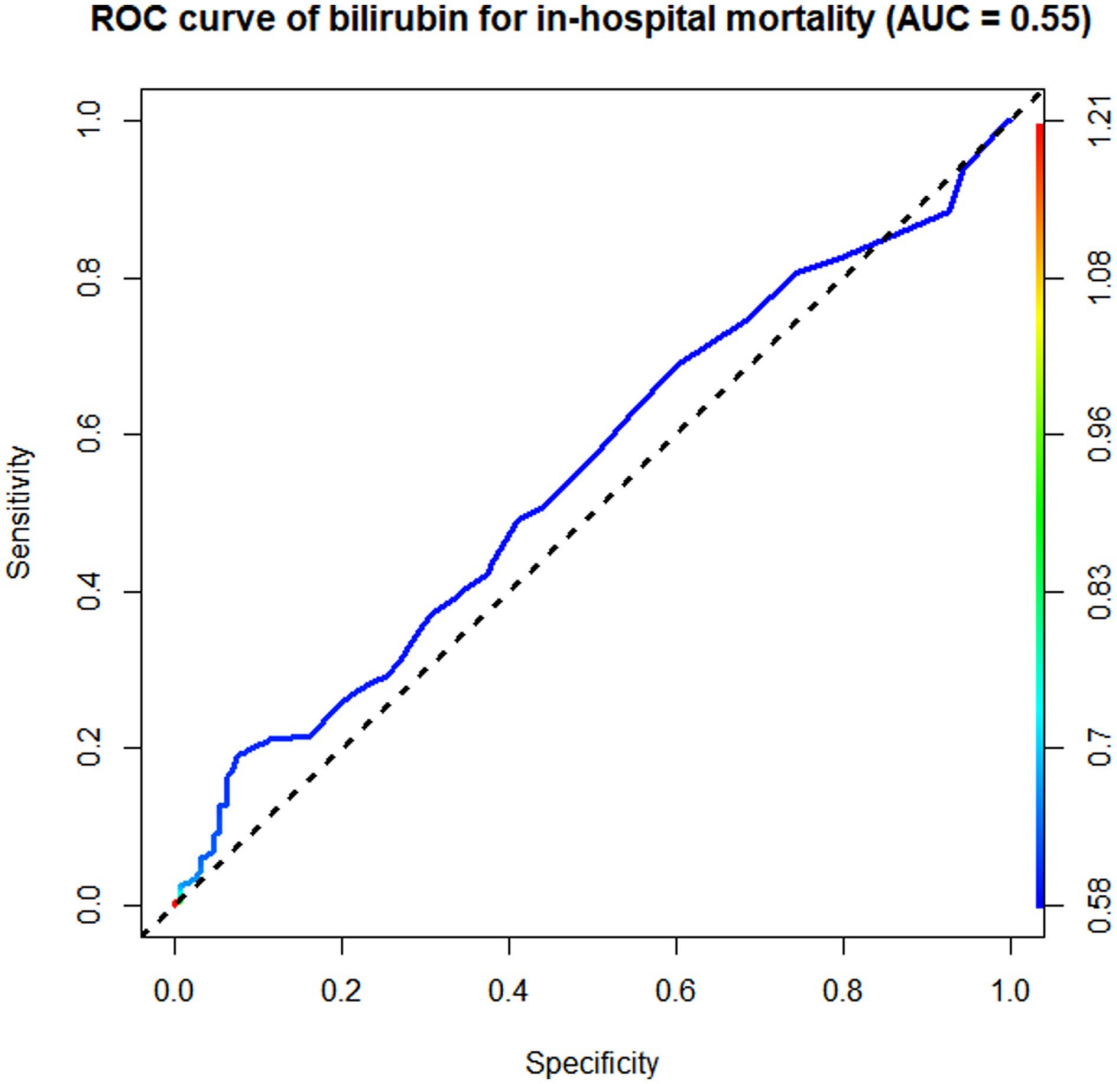
Receiver operating characteristic (ROC) curve of bilirubin.

According to the ROC curve analysis results, the AUC of in-hospital mortality in patients with AIS predicted by the SII was 0.65 (95% CI 0.62–0.68), the AUC of the NLR was 0.64 (95% CI 0.61–0.67), the AUC of the PLR was 0.60 (95% CI 0.53–0.67), and the AUC of total bilirubin was 0.55 (95% CI 0.52–0.58). The SII had a better discriminative ability for predicting in-hospital mortality in patients with AIS than the NLR, PLR, and total bilirubin. Overall, the SII had a modest discriminative ability for predicting in-hospital mortality (Figures 3–5).

## Discussion

This study revealed a positive correlation between the in-hospital mortality of patients with AIS and the SII, but this correlation was not linear. The overall in-hospital mortality rate of patients with AIS was 12.31%, which is similar to the value of 13.9% from the Get With The Guidelines-Stroke database of the American Heart Association (18). We found that (1) a high SII was independently associated with a high risk of in-hospital mortality in patients with AIS; (2) hospitalized patients with AIS with a high SII were less likely to survive than patients with a low SII; (3) SII had a modest discriminative ability for forecasting in-hospital mortality in patients with AIS; and (4) the SII was marginally superior to the NLR and significantly better than the PLR and total bilirubin in forecasting in-hospital mortality in patients with AIS.

We also found that the values for temperature, anion gap, blood glucose, and SAPS II were higher among patients with a high SII, indicating that these indicators are strongly associated with poor outcomes in patients with AIS, as demonstrated in several previous studies. For example, a previous study showed that elevated body temperature was associated with increased mortality and poor functional outcome in patients with AIS (19). Another study showed that patients with an elevated plasma anion gap had worse clinical outcomes and were at a greater risk of in-hospital mortality (20). Moreover, hyperglycemia on admission has been linked to worse post-stroke outcomes (21). SAPS II, which was created to assess disease severity in patients in the ICU, is also linked to poor outcomes in patients with AIS (22).

The pathophysiological process after AIS is complex, and inflammation plays a key role, starting in the vascular compartment immediately after arterial blockage (23). Neutrophils, which are the earliest cells to react during ischemic stroke and are clinically associated with a poor functional outcome, begin to enter the brain parenchyma 12 h after stroke onset, causing neuronal death by producing elastase, matrix metalloproteinase-1, interleukin-7β, and reactive oxygen species. This in turn destroys the blood–brain barrier and induces damage to the ischemic area (5, 24). Additionally, neutrophils express inducible nitric oxide synthase, which is an enzyme that catalyzes the generation of nitric oxide and causes bigger infarcts during middle cerebral artery occlusion (25). Therefore, the increase in neutrophils is a key mediator of ischemic brain damage. Platelets interact with neutrophils and are key players in thrombotic inflammation and stroke pathogenesis (26). Similar to neutrophils, activated platelets interact with the endothelium and release mediators that promote inflammation after stroke, aggravating the inflammatory immune response (27). A previous study revealed that platelet P-selectin and glycoprotein Ib, which bind neutrophil P-selectin glycoprotein-1 and MAC-1 (CD11b/CD18), facilitate this interaction (28). Lymphocytes also play an important role in the inflammatory response in AIS, although the pathogenic role of lymphocytes is controversial. T cells play a key role in the exacerbation of ischemic brain injury (5); however, regulatory T cells are a major protective modulator of post-ischemic brain injury (29). For example, in a mouse experiment, regulatory T cell-treated mice had smaller infarcts and improved neurological function after stroke (30).

The SII, which is a novel comprehensive inflammatory index, is calculated based on three inflammatory immune cell types (lymphocytes, neutrophils, and platelets) that reflect the balance between the immunological and inflammatory states of the host. The SII was initially used to predict tumor prognosis and identify patients at a high risk of death (31). The SII has reportedly been linked to increased disease severity and a poor prognosis in various illnesses and could be used to anticipate fatality in patients with various cancers, heart failure, and cardiovascular disease (32). Thus far, the value of the SII in cerebrovascular illnesses has been demonstrated in several studies. For example, studies by Zhou et al. (33) and Wang et al. (34) showed that a high SII increases the risk of death from AIS. Moreover, Zhang’s study showed that a high SII may adversely affect carotid plaque vulnerability. Specifically, patients with fragile plaques with burst fibrous caps may experience a considerable impact, which might worsen the severity of AIS (35). A previous study based on the MIMIC-IV database showed that a high SII increased 30-day all-cause mortality (36). Chen investigated the difference between four inflammatory immune markers in predicting the outcome of patients with ischemic stroke (37). Moreover, in a meta-analysis, high SII was strongly associated with poor ischemic stroke outcomes and a high mortality rate (38). However, more research is needed to ascertain the association between the SII and in-hospital mortality in patients with AIS.

According to our literature review, this may be the first study to assess the relationship between markers of both inflammation and oxidative stress and in-hospital mortality in patients with AIS. Focusing on the SII, we assessed the predictive power of four markers (SII, NLR, PLR, and total bilirubin) simultaneously. The study demonstrated that an elevated SII was significantly associated with the risk of in-hospital mortality in patients with AIS. However, whether the SII predicts in-hospital mortality in patients with AIS better than the NLR is less reported. We found that the SII was a greater prognosticator of in-hospital mortality than the NLR (AUC 0.65 vs. 0.64, respectively). Therefore, compared with the NLR, the SII might be a more reasonable and valid reflection of the overall change and regression status of the immune system in patients with stroke. The NLR mainly suggests inflammatory injury, the PLR demonstrates an impact on hemostasis and thrombosis, and SII can be thought of as a combination of the NLR and PLR. Therefore, an elevated SII may reflect thrombosis, the inflammatory response, and the adaptive immunological response (11).

In this study, we adjusted for the mixed effects of several factors based on rigorous study principles. We confirmed that the SII measured within 24 h of admission was significantly associated with adverse clinical outcomes. These data revealed an independent relationship between the SII and the risk of in-hospital mortality in patients with AIS. Few studies have explored the importance of the SII in predicting in-hospital mortality in patients with AIS. Our findings are based on a large sample; therefore, data from a more diverse group of patients in the clinical setting were included. The findings imply that the SII could be utilized to forecast prognosis in patients with AIS. According to this study, patients with an SII of >1,232 (Q2 group) had a considerably higher chance of dying in the hospital than patients with a low SII (Q1 group). Therefore, for patients with AIS with an elevated SII, there is a need to clarify the cause of the high SII and to provide more appropriate treatment. The value of the SII for identifying high-risk subgroups with AIS needs to be further explored in the future to provide more valuable guidance for early targeted treatment. In addition, blood analysis is available at the time of admission. As such, the SII is readily available, can be performed at no additional cost, and has relatively high patient compliance. The SII can therefore be used as a supplement to blood gas analysis.

## Conclusion

In this study, the in-hospital mortality of patients with AIS and the SII were positively correlated, but not linearly. A high SII was associated with a worse prognosis in patients with AIS. The SII had a modest discriminative ability for predicting in-hospital mortality in patients with AIS. The SII was slightly better than the NLR and significantly better than the PLR and total bilirubin at forecasting in-hospital mortality in patients with AIS.

### Limitations

This study has several limitations that should be noted. First, this study did not categorize the patients who died based on whether they had undergone surgery. Therefore, future research should analyze different patient subgroups. Second, this was a single-center study, so the study could contain selection bias. Third, this study lacked some blood indicators, including interleukin, high-sensitivity C-reactive protein, and other cytokines. Finally, only the in-hospital mortality of patients with AIS was studied. As such, additional research is needed to determine how useful the SII is for predicting the long-term prognosis of patients with AIS.

## Data availability statement

The original contributions presented in the study are included in the article/Supplementary material, further inquiries can be directed to the corresponding authors.

## Author contributions

XH developed the study protocol’s design, gathered and analyzed the data, and wrote the majority of the original draft text for publication. JL and WH provided support for editing the manuscript. A specialist clinical analysis was provided by YL and XG. The data were examined and the data confirmation was finished by JZ and YG. All authors contributed to the article and approved the submitted version.

## Funding

The authors received funding for this project from the Natural Science Fund of Liaoning Province (2021-MS-282) and the National Backbone of TCM Innovation [TCM Education Letter (2019) No. 128].

## Conflict of interest

The authors declare that the research was conducted in the absence of any commercial or financial relationships that could be construed as a potential conflict of interest.

## Publisher’s note

All claims expressed in this article are solely those of the authors and do not necessarily represent those of their affiliated organizations, or those of the publisher, the editors and the reviewers. Any product that may be evaluated in this article, or claim that may be made by its manufacturer, is not guaranteed or endorsed by the publisher.
