# Supplementary material for: Prognostic value of inflammatory markers for in-hospital mortality in intensive care patients with acute ischemic stroke: a retrospective observational study based on MIMIC-IV
Source: Front Neurol. 2023 Jun 8;14:1174711. doi: 10.3389/fneur.2023.1174711 (PMC10285211; doi:10.3389/fneur.2023.1174711)
Supplement: Supplementary file 1 [file Table_1.DOCX]

**Table S1 Number of observations after each selection procedure**

| Step | Criteria | Number excluded | Number of remaining observations |
| --- | --- | --- | --- |
| 1 | Total subjects admissions included in MIMIC-IV database | - | 255106 |
| 2 | Total subjects ICU admissions included in MIMIC-IV database | - | 53150 |
| 3 | Include patients on admission diagnosed with AIS (ICD-9 code and ICD-10 code between) and stay in ICU | - | 1605 |
| 4 | Exclude if patients with missing vital signs data information | - | 1601 |
| 5 | Exclude if patients with missing neutrophils,lymphocytes and platelet counts data information | - | 463 |

**Table S2 Missing number (%) for characteristics**

| Characteristics | Missing number (%) |
| --- | --- |
| Demographics |  |
| Age | 0 |
| Gender | 0 |
| Ethnicity | 0 |
| Marital status | 0 |
| Vital signs |  |
| HR | 0 |
| SBP | 0 |
| DBP | 0 |
| MBP | 0 |
| RR | 0 |
| T | 0 |
| SpO2 | 0 |
| Comorbidities |  |
| diabetes | 0 |
| m1alignant_cancer | 0 |
| myocardial infarction | 0 |
| dementia | 0 |
| renal_disease | 0 |
| chronic_pul1onary_disease | 0 |
| congestive_heart_failure | 0 |
| Laboratory parameters | 0 |
| aniongap | 0 |
| bicarbonate | 0 |
| bun | 0 |
| creatinine | 0 |
| chloride | 0 |
| glucose | 0 |
| [bilirubin](javascript:;) | 0 |
| hematocrit | 0 |
| hemoglobin | 0 |
| potassium | 0 |
| PT | 0 |
| sodium | 0 |
| wbc | 0 |
| platelet | 0 |
| neutrophils | 0 |
| lymphocytes | 0 |
| SOFA | 0 |
| SAPSII | 0 |
| ICU LOS | 0 |
| In-hospital LOS | 0 |
| In-hospital mortality | 0 |
